# Modeling Urothelial Carcinoma and Immunotherapy Response Using Organoid Platforms: From Epithelial Tumor Organoids to Multilineage Systems

**DOI:** 10.3390/cancers18091338

**Published:** 2026-04-23

**Authors:** Jorge O. Múnera

**Affiliations:** Department of Regenerative Medicine and Cell Biology, Medical University of South Carolina, Charleston, SC 29425, USA; munera@musc.edu

**Keywords:** organoids, assembloids, tumor organoids, urothelial cancer, induced pluripotent stem cells

## Abstract

Cancer immunotherapy has transformed oncology, including the management of urothelial carcinoma, yet response rates remain limited and mechanisms of resistance are incompletely understood. At the same time, recent initiatives from the National Institutes of Health and the United States Food and Drug Administration have emphasized the development of standardized human organoid platforms to improve preclinical modeling and reduce reliance on traditional animal systems. Urothelial cancer provides a compelling context in which to advance these efforts, given its established responsiveness to Bacillus Calmette–Guérin and immune checkpoint blockade and its marked heterogeneity in clinical outcomes. Here, we review current organoid-based platforms for modeling bladder cancer immunotherapy, including patient biopsy-derived epithelial tumor organoids, air–liquid interface cultures that retain endogenous stromal and immune components, engineered bladder cancer assembloids that reconstruct defined multicellular circuits, and induced pluripotent stem cell-derived urothelial organoids that offer renewable multilineage systems. For each approach, we outline strengths, technical constraints, and limitations in scalability, immune fidelity, and genetic matching. We conclude by discussing the critical challenges that must be addressed—including benchmarking to patient tumors, reproducibility across laboratories, and standardized validation metrics—to enable regulatory acceptance and clinical translation of organoid-based immunotherapy testing.

## Simple Summary

This review examines how organoid platforms are being used to model urothelial carcinoma and its response to immunotherapy. It argues that no single model is sufficient: epithelial tumor organoids are useful for tumor-intrinsic biology, air–liquid interface cultures better preserve native immune and stromal components, assembloids allow controlled reconstruction of multicellular interactions, and pluripotent stem cell-derived urothelial organoids offer a renewable multilineage system. The paper emphasizes that immunotherapy response in bladder cancer depends on both tumor-intrinsic features and the tumor microenvironment, so models that exclude stroma and immune cells are limited. It concludes that the field needs rigorous benchmarking against patient tumors, improved reproducibility, and standardized validation metrics before these systems can be reliably used for translational studies, regulatory acceptance, or clinical prediction.

## 1. Introduction and Historical Perspective

Urothelial carcinoma, most commonly arising in the bladder, represents a major global cancer burden. It is among the ten most frequently diagnosed cancers worldwide, with nearly six hundred thousand new cases and more than two hundred thousand deaths reported annually [1]. In the United States alone, bladder cancer accounts for approximately eighty-four thousand new diagnoses and seventeen thousand deaths each year, with a marked male predominance [2]. This substantial incidence, combined with high recurrence rates and long-term morbidity, underscores the need for durable and mechanistically informed therapeutic strategies.

Bacillus Calmette–Guérin therapy represents the earliest successful example of cancer immunotherapy and remains the standard adjuvant treatment for high-risk non–muscle-invasive bladder cancer. BCG is a live attenuated strain of Mycobacterium bovis originally developed as a tuberculosis vaccine and later repurposed for intravesical cancer therapy following the seminal observations by Morales and colleagues in the mid-1970s [3]. Mechanistically, BCG activates innate immune pathways through pattern-recognition receptors, leading to the recruitment of neutrophils, macrophages, dendritic cells, and natural killer cells. This innate activation is accompanied by a strong Th1-polarized cytokine milieu characterized by interferon gamma, tumor necrosis factor alpha, and interleukin-12 [4,5]. Antigen presentation subsequently drives adaptive immune responses involving CD4-positive and CD8-positive T cells that mediate tumor cell killing. While BCG can induce durable remission in a substantial fraction of bladder cancer patients, resistance and relapse remain common [6].

Building on the immunogenic nature of urothelial carcinoma, immune checkpoint inhibitors targeting programmed cell death protein 1 (PD-1) and programmed death-ligand 1 (PD-L1) have become central to the treatment of advanced disease [7,8,9]. Multiple PD-1 and PD-L1 blocking antibodies have been approved for metastatic and muscle-invasive bladder cancer based on the durable clinical benefit observed in pivotal trials [7,10,11,12]. Although these agents can induce long-lasting responses in a subset of patients, objective response rates to monotherapy typically remain limited to approximately fifteen to twenty-five percent [7,9,10,13].

Clinical and translational studies have demonstrated that response heterogeneity reflects both tumor-intrinsic features, including tumor mutational burden, neoantigen load, and defects in antigen presentation, as well as extrinsic features of the tumor microenvironment such as immune cell composition, cytokine signaling, and stromal architecture [7,14,15,16]. Therefore, in vitro models that faithfully recapitulate both tumor intrinsic properties and critical components of the tumor microenvironment are required to enable mechanistic dissection of how these interacting features determine immunotherapy sensitivity, primary resistance, and acquired immune escape. Herein, we review the emerging use of urothelial cancer organoid models as platforms for immunotherapy research, highlighting current progress in modeling tumor–immune interactions, therapeutic response, and resistance, and outlining the technical, biological, and regulatory challenges that must be addressed for these systems to serve as standardized platforms for preclinical drug screening and eventual regulatory evaluation.

## 2. Patient-Derived Urothelial Carcinoma Organoids

**Figure 1:**
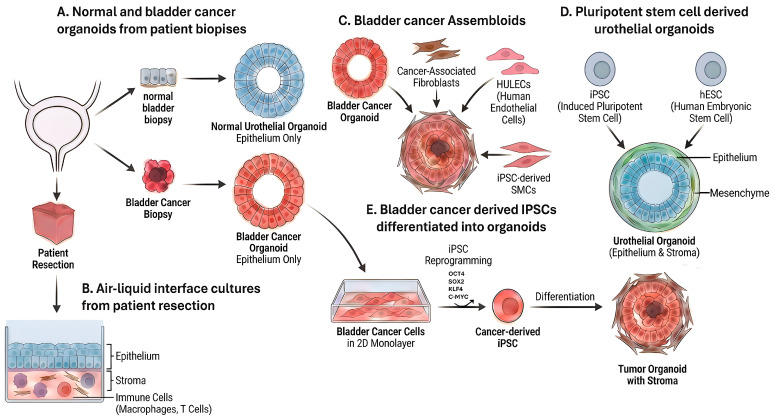
Organoid models of normal and malignant human urothelium. (**A**) Patient-derived epithelial organoids established from normal bladder biopsies and bladder cancer resections. (**B**) Air–liquid interface cultures generated from patient tumor resections that preserve epithelial, stromal, and immune components, including macrophages and T cells, within native-like tissue architecture. (**C**) Bladder cancer assembloids produced by combining tumor organoids with defined stromal and vascular components, including cancer-associated fibroblasts, endothelial cells such as HULECs, and induced pluripotent stem cell-derived smooth muscle cells, to model multicellular tumor–stroma interactions. (**D**) Pluripotent stem cell-derived urothelial organoids containing co-developing epithelial and mesenchymal compartments generated from human embryonic stem cells or induced pluripotent stem cells, providing renewable multilineage urothelial tissue. (**E**) Cancer-derived induced pluripotent stem cell route in which bladder cancer cells expanded in two-dimensional culture are reprogrammed into induced pluripotent stem cells and subsequently differentiated into urothelial organoids containing tumor epithelium and stromal components.

Organoids are three-dimensional (3D) structures grown from stem cells that model the cellular complexity and self-organization of native organs [17,18,19]. They have become a cornerstone of biomedical research, enabling studies of human development, physiology, and disease. In bladder cancer, patient-derived tumor organoids (PDOs, also called tumoroids) have emerged as valuable models that preserve the histological and genetic characteristics of the patient’s tumor (Figure 1A) [20,21,22,23]. Bladder cancer organoids can be established from tumor biopsies and these bladder tumoroids maintain the genetic and transcriptional heterogeneity of urothelial carcinoma: for instance, organoids exhibited both luminal and basal phenotypes corresponding to The Cancer Genome Atlas (TCGA) molecular subtypes, and harbored common mutations such as TP53 and FGFR3 akin to their parental tumors.

**Figure 2:**
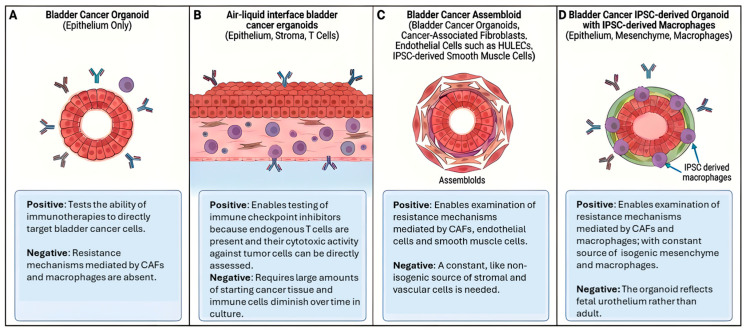
Pros and cons of different bladder cancer organoid systems for modeling the response to immunotherapies. Strengths and weaknesses of (**A**) bladder cancer epithelial organoids composed of tumor epithelium only, (**B**) air–liquid interface bladder cancer cultures containing epithelium, stroma, and endogenous immune cells, including T cells, (**C**) bladder cancer assembloids incorporating bladder cancer organoids with cancer-associated fibroblasts, HULECs and iPSC derived smooth muscle cells (SMCs) and (**D**) bladder cancer induced pluripotent stem cell-derived organoids containing epithelium, mesenchyme, and induced pluripotent stem cell-derived macrophages.

Although bladder cancer organoids can maintain the molecular and histological features of the tumor from which they are derived, one study found that nearly 2/3rds of organoids switch from luminal to basal phenotype in vitro. However, these organoids reverted back to a luminal state in xenografts, and then reacquired basal features upon re-derivation, indicating a largely reversible, culture-associated basal transition [20]. These results suggest that the stromal components may play a critical role in maintaining this molecular subtype in a subset of tumor organoids. In addition, standard organoid cultures lack the non-epithelial components of the tumor microenvironment, which are important to model since they can contribute to therapy resistance (Figure 2A) [14,15,16].

A recently described immunocompetent bladder cancer organoid platform was developed to enable evaluation of therapeutic responses within a preserved tumor–immune context [24]. In this system, patient tumor tissue is dissociated into single cells and cultured in low-adhesion suspension conditions without extracellular matrix scaffolds, allowing tumor cells to self-organize into three-dimensional structures while retaining endogenous tumor-infiltrating lymphocytes. Maintenance of these immune populations is supported through supplementation with factors that promote T cell survival and activity, and matched peripheral immune cells can be incorporated when endogenous immune infiltration is limited. These matrix-free organoids preserve key histologic, genomic, and molecular features of the parental tumors together with functional T cell populations, enabling direct assessment of tumor–immune interactions and therapeutic response. Using this approach, the authors identified chemotherapeutic agents that modulate anti-tumor immune activity and influence responses to checkpoint blockade in a patient-specific manner, highlighting the potential of suspension-based immunocompetent organoid systems as scalable platforms for modeling combined chemoimmunotherapy and treatment heterogeneity.

In relation to immunotherapy, one of the few papers that begins to address the clinical relevance tumor organoids directly is a study that describes the generation of urinoids [23]. This study demonstrated that urine-derived bladder cancer organoids could be established noninvasively in 55 percent of patients (12 urinoid lines from 22 patient biopsies) and that these cultures broadly recapitulated the histology and molecular features of the parental tumors. In a particularly informative longitudinal case, they generated organoids before and after immunotherapy, identified therapy-associated genomic changes in both urine-derived and tissue-derived lines, and found concordant post-treatment sensitivity to vincristine and vinblastine. That makes this an important proof of concept for longitudinal monitoring and therapy adaptation. At the same time, it is not full clinical validation, because the strongest treatment-matching evidence comes from essentially one patient-level example rather than a prospective cohort study linking organoid predictions to patient outcomes.

## 3. Air–Liquid Interface Cultures

Air–liquid interface cultures are a three-dimensional tumor culture approach designed to preserve the structural and cellular complexity of native tumors more faithfully than conventional epithelial-only organoid systems [25]. This method was originally developed for the growth of gastrointestinal tissues [26]. In this method, mechanically dissociated tumor fragments are embedded within a collagen matrix on a permeable support and maintained with media supplied from below while the upper surface remains exposed to air (Figure 1B). This configuration allows oxygenation and nutrient exchange while maintaining tissue architecture. Because tumor fragments are not enzymatically dissociated into single epithelial cells before culture, this approach preserves multicellular organization and enables short term maintenance of the native tumor microenvironment. As a result, air–liquid interface systems have been used to generate ex vivo tumor cultures from multiple cancer types for mechanistic studies and therapeutic testing [25].

A central advantage of air–liquid interface cultures is their ability to retain non-epithelial components of the tumor microenvironment that are typically lost in standard submerged organoid cultures [25]. These cultures can preserve endogenous immune populations, including tumor infiltrating lymphocytes and myeloid cells, along with fibroblasts and other stromal elements that shape tumor behavior in vivo. Preservation of these compartments allows examination of tumor immune interactions and has enabled direct testing of immunotherapies such as checkpoint blockade in a setting that maintains native immune repertoires and tumor antigen presentation. In addition to maintaining immune and stromal components, these cultures retain key genetic and histologic features of the original tumor and can be established relatively rapidly, making them useful for short term functional studies and patient-specific therapeutic testing. Their ability to maintain multicellular architecture and heterogeneity provides a more physiologically relevant system than epithelial-only organoids for questions related to tumor microenvironment signaling and immune response.

Despite these strengths, air–liquid interface cultures have important limitations (Figure 2B). Although they initially preserve immune and stromal components, these populations decline over time, which constrains the duration over which intact tumor microenvironment interactions can be studied. For this reason, the assays for anti-PD-1-dependent human TIL activation and tumor cell killing were limited to 7 days of culture. Long-term maintenance of a fully functional immune compartment remains difficult, and prolonged culture often results in progressive loss or functional alteration of non-epithelial cells. In addition, these cultures do not incorporate circulating immune elements or systemic influences that contribute to tumor evolution and therapeutic response in vivo, limiting their ability to fully model complex immune dynamics such as recruitment of peripheral immune cells. Technical complexity and variability in tissue quality can also affect reproducibility across samples. As a result, while air–liquid interface systems provide a more complete representation of tumor architecture and microenvironment than conventional organoid cultures, they remain best suited for short to intermediate term studies of tumor–stroma and tumor–immune interactions rather than long term modeling of tumor evolution.

## 4. Assembloids

Another approach for introducing stromal elements to organoids is the generation of assembloids. Assembloids are generated by combining tumor organoids with stromal cell types. In one study, human bladder cancer organoids were combined with cancer-associated fibroblasts, human lung microvascular endothelial cells (HULECs) and induced pluripotent stem cell-derived smooth muscle cells (Figure 1C) [27]. These assembloids were used to identify a mechanistically specific, progression-relevant crosstalk circuit: stromal-derived bone morphogenetic protein (BMP) signaling induced tumor cells to upregulate the pioneer transcription factor FOXA1, which then drove epigenetic reprogramming and altered tumor-state identity. This work highlighted a FOXA1–BMP–Hedgehog signaling axis linking cancer and stroma that helps govern tumor plasticity—an interaction that is difficult to discover or interpret in pure epithelial cultures that lack organized stromal context.

In addition to co-culture of tumor organoids with CAFs and endothelial cells, several co-culture systems have been developed to integrate immune cells. In one study, bladder cancer organoids were combined with autologous or engineered lymphocytes to model antigen-specific immune killing. Bladder tumor organoids and co-cultured with MUC1-targeted CAR-T cells, demonstrated selective cytotoxicity toward antigen-positive organoids and recapitulated tumor antigen heterogeneity observed in patient samples [28]. This work illustrates how organoid–immune co-cultures can serve as high-throughput platforms for evaluating cellular immunotherapies in a patient-specific manner.

The assembloid strategy is conceptually powerful, but two practical weaknesses repeatedly limit throughput and interpretability (Figure 2C). First, multi-lineage synchronization is technically demanding. Each compartment (epithelial tumor organoids, fibroblasts/CAFs, smooth muscle, endothelial cells, and immune populations) typically requires different culture conditions, expansion kinetics, maturation states, and passaging schedules. Assembling them into a stable, reproducible construct requires aligning these variables within a narrow window of compatibility. In practice, this synchronization burden is often the dominant barrier to scale, standardization, and long-term experiments. Second, assembloids commonly rely on non-isogenic cellular sourcing, which can confound mechanistic conclusions—especially for immune-focused questions. In addition, the limited lifespan of primary CAFs and the variability of freshly derived immune populations make it difficult to carry out durable, patient-matched assembloid studies that remain stable across repeated perturbations and extended treatment timelines.

## 5. Pluripotent Stem Cell-Derived Urothelial Organoids

Human pluripotent stem cells (which include embryonic stem cells and induced pluripotent stem cells and will be herein referred to as hPSCs) provide a renewable platform with the capacity for indefinite self-renewal and the ability to differentiate into virtually any tissue type in the human body [29,30], enabling scalable modeling of human development and disease. Although multiple studies have reported the generation of urothelial tissue from hPSCs [31,32,33,34], these urothelial cells have largely been produced using reductionist differentiation approaches that do not recapitulate the multicellular architecture of the native bladder. In addition, these urothelial cultures lack a stromal component.

Recently, our lab developed a method for generating human urothelial organoids that contain co-developing stromal cells including fibroblasts and endothelial cells (Figure 1D) [35]. Because these organoids are derived from hPSCs, they provide a renewable and scalable source of urothelial tissue together with matched stromal components, enabling sustained production of urothelial fibroblasts and endothelial cells without dependence on primary tissue. Because induced pluripotent stem cells (iPSCs) can be generated from essentially any starting cell type, primary bladder cancer cells or patient-derived bladder cancer organoids can in principle be reprogrammed into iPSCs that carry the tumors mutations. Differentiating the tumor derived iPSCs into urothelial organoids would then be expected to preserve the tumor genomic background while simultaneously producing stromal compartments within the same pluripotent stem cell-derived organoid system, creating a renewable source of genetically matched tumor epithelium together with tumor matched stroma (Figure 1E).

Even if reprogramming bladder cancer cells or tumor organoids into iPSCs and differentiating them into urothelial organoids proves feasible, this strategy presents important limitations (Figure 2D). Organoids derived from pluripotent stem cells typically resemble fetal stage tissue, which may not fully capture the differentiated state of adult urothelium. However, studies using hPSC-derived human intestinal organoids (HIOs), which are derived from the same mid/hindgut progenitors as ipSC-derived urothelial organoids (HUOs), have shown that prolonged in vitro culture can promote maturation toward a more adult-like phenotype [36]. HUOs undergo similar maturation as HIOs in early stages of culture, so one would expect that extended culture would allow HUOs to mature into adult-like organoids. A second limitation is that stromal lineages generated from tumor-derived iPSCs would carry the same oncogenic mutations as the epithelial compartment, which does not reflect the genetic composition of stromal cells in native bladder tumors where most cancer associated mutations are epithelial restricted. As a result, fully leveraging this platform for modeling tumor microenvironment interactions will likely require parallel derivation of iPSCs from non-transformed patient-matched cells such as peripheral blood mononuclear cells to generate genetically normal stromal and immune compartments that can be combined with tumor-derived organoids. This would enable controlled reconstruction of epithelial stromal interactions in an isogenic yet physiologically relevant context.

## 6. Translational Implications and Future Directions

The expanding toolkit of urothelial carcinoma models—including patient-derived tumor organoids, air–liquid interface cultures, assembloids, and possibly hPSC-derived urothelial organoids—should not be viewed as competing platforms, but as complementary systems tailored to address distinct biological questions (Table 1). Epithelial patient-derived organoids are well-suited for interrogating tumor-intrinsic genomics and therapeutic sensitivity. Air–liquid interface cultures demonstrate that preservation of native tumor architecture and immune components is achievable, although scalability remains limited. Assembloids enable controlled reconstruction of epithelial–stromal and epithelial–immune signaling circuits. hPSC-derived systems uniquely provide renewable, multilineage platforms capable of generating matched epithelial, stromal, endothelial, and potentially immune compartments. The central translational goal is to develop in vitro systems that most faithfully model the patient tumor within its native microenvironment. Achieving this objective will likely require integration of these complementary platforms together with bioengineering strategies such as microfluidic systems that enable controlled immune trafficking, cytokine gradients, and physiologic drug exposure.

Recent initiatives from the National Institutes of Health and the U.S. Food and Drug Administration have placed increasing emphasis on the development of standardized human organoid platforms for preclinical testing and therapeutic evaluation as part of their New Approach Methodology (NAM) initiative. The FDA evaluates NAMs using four core criteria, each with specific requirements [37]. First, the context of use requires a precise definition of what decision the model will inform, such as dose selection, safety risk assessment, mechanistic interpretation of an observed effect, or justification for not using an animal model; the model must directly address a defined regulatory question rather than provide general biological insight. Second, human biological relevance requires demonstration that the system contains the appropriate human cell types, architecture, and functional properties to recapitulate the physiology being interrogated, along with measurable endpoints that reflect clinically meaningful biology rather than descriptive features alone. Third, technical characterization requires detailed documentation of the system, including cell source and identity, culture conditions, assay stability, reproducibility across batches, performance metrics such as sensitivity and specificity, and identification of sources of variability such as donor differences. Fourth, fit-for-purpose requires evidence that the model can reliably inform decision-making, either by replacing an existing method, filling a defined data gap, or supporting a weight-of-evidence approach, with clear acknowledgment of its limitations and benchmarking against existing approaches when possible.

Within this framework, rigorous benchmarking of organoid systems against matched primary tumors will be essential. Models must be evaluated at the genomic, transcriptional, cellular, and functional levels to ensure that they accurately preserve tumor subtype identity, stromal architecture, and immune responsiveness observed in vivo. At the genomic level, organoid systems should retain the same mutational signature as the tumor from which they were derived. At the transcriptional level, they should preserve the gene expression profile of the original tumor. At the cellular level, organoid systems should maintain not only the same cell types present in the original tumor, but also their spatial organization, so that cell positioning mirrors that observed in vivo. At the functional level, immunotherapies should be effective at killing tumor cells and should induce cytokine secretion patterns consistent with those expected in vivo. Without systematic benchmarking to patient tissue and clinical response, the predictive value of organoid-based immunotherapy testing will remain uncertain. Establishing validated reference standards and shared analytical pipelines will therefore be critical for widespread adoption in both academic and regulatory settings.

A central challenge for translation is reproducibility across laboratories and across patient samples. Variability in primary stromal isolation, immune cell sourcing, and culture conditions continues to limit scalability and standardization. Integration of patient-derived tumor organoids with non-tumor iPSC-derived stromal and immune compartments generated from the same patient offers a practical path forward. Unlike primary cancer-associated fibroblasts or peripheral immune populations, which are finite and often difficult to repeatedly obtain, iPSC-derived lineages would provide renewable and genetically stable sources of fibroblasts [35], endothelial cells [38], macrophages [38], and T cells [39,40] to incorporate into assembloids. This renewable supply of cells would enable repeated perturbation studies, longitudinal analyses, and cross-laboratory comparisons under controlled conditions.

A multi-pronged strategy that combines tumor organoids with patient-matched iPSC-derived stromal and immune compartments will be required to build models suitable for mechanistic studies, therapeutic screening, and regulatory applications. Generating these components from the same patient will allow reconstruction of epithelial–stromal–immune interactions within a shared genetic background while preserving experimental scalability. As protocols become standardized and broadly disseminated, such integrated systems have the potential to support reproducible drug testing, identification of predictive biomarkers, and ultimately the development of organoid platforms capable of informing immunotherapy selection and therapeutic development for patients with urothelial carcinoma.

## Figures and Tables

**Table 1:** Detailed comparison of organoid systems.

Platform	Structural Complexity	Source of Immune Cells	Immune Fidelity	Source of Tissue	Scalability	Immune Modeling Functions	Key Limitations
Epithelial Tumor Organoids	Epithelial only	Not applicable	Not applicable	Direct from patient fine needle biopsies	High; rapid expansion possible	Not applicable	Lacks microenvironment; phenotype drift in vitro
Air–Liquid Interface (ALI)	Preserves native architecture, includes stroma and immune cells	Endogenous from patient tissue; declines over time	Moderate; retains endogenous repertoire short-term	Direct from patient tumor resections	Low to moderate; tissue quality-dependent, short-term viability	Antigen presentation, native T cell activation, short-term myeloid function	Immune decline over time, limited duration
Assembloids	Engineered assembly of epithelial, stromal, vascular, immune components	Exogenous cells introduced by co-culture with epithelial tumor organoids, e.g., CAR-T, autologous lymphocytes	High in concept but variable; limited by sourcing and lifespan	Non-isogenic mix; tumor organoids + third-party components	Low; technical burden for synchronization and reproducibility	CAR-T cytotoxicity, immune-epithelial signaling pathways	Difficult to scale, non-isogenic limitations
iPSC-Derived Organoids	Multilineage from single iPSC source, includes stromal and vascular cells	Theoretical; possible via isogenic derivation from the same source iPSCs	Currently limited to monocytes although T cell generation possible; immune components may be immature	Potential for isogenic reconstruction via iPSC reprogramming	Moderate; renewable but complex differentiation needed	Under development; potential for engineered immune interfaces	Fetal-like phenotype, oncogenic stroma unless corrected

## Data Availability

No new data were created or analyzed in this study. Data sharing is not applicable to this article.
